# In memoriam

**Published:** 2025-12-10

**José Moreno-Montoya** 1980-2025 Comité Editorial

**Figure:**
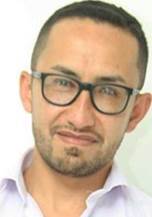

El Comité Editorial de *Biomédica* comunica con profundo pesar a sus lectores, colaboradores y a la comunidad científica en general, el fallecimiento del doctor José Moreno-Montoya (1980-2025), quien se desempeñó con ejemplar dedicación como editor asociado de esta revista desde el número 3 del 2017. Su deceso, ocurrido en Bucaramanga (Santander) el pasado fin de semana, representa una pérdida de enorme magnitud para el ámbito académico y editorial del país.

El doctor Moreno-Montoya se distinguió por un ejercicio editorial estricto guiado por la oportunidad, la objetividad, la justicia y el respeto. Cada una de sus intervenciones en el proceso de evaluación científica se sustentó en principios éticos sólidos y en una comprensión profunda de los métodos de investigación, lo que garantizó a los autores y revisores un trato equilibrado, transparente y altamente profesional.

Su notable capacidad para analizar con rigor los aspectos metodológicos de los manuscritos, así como para identificar fortalezas, debilidades y oportunidades de mejora, contribuyó de manera decisiva a elevar los estándares de calidad científica de *Biomédica.* Su dominio en estadística, epidemiología y salud pública enriqueció el fondo y la forma de los artículos publicados en la revista.

La serenidad, claridad y precisión de sus conceptos editoriales, siempre expresados con lenguaje respetuoso y constructivo, fueron un ejemplo permanente de excelencia y compromiso con el rigor académico. A ello se sumó su espíritu colaborativo y su profundo respeto por la diversidad de perspectivas científicas, cualidades que fortalecieron el trabajo colectivo del Comité Editorial. El doctor Moreno-Montoya promovió activamente el debate informado, la reflexión crítica y la adopción de decisiones orientadas a preservar la integridad editorial, dejando un legado que seguirá orientando esta labor por muchos años.

En el plano humano, su partida deja un vacío inmenso. Quienes tuvimos el privilegio de conocerlo y trabajar a su lado valoramos no solo su inteligencia y disciplina intelectual, sino también la nobleza de su carácter. Su disposición permanente para acompañar, escuchar y orientar, su calidez en el diálogo cotidiano y su generosidad sin ostentación hicieron de él un amigo excepcional, cuya memoria permanecerá viva en la historia de *Biomédica* y en la vida de quienes compartimos parte de su camino.

El Comité Editorial expresa sus más sentidas y respetuosas condolencias a sus familiares, allegados y amigos. A ellos les ofrecemos nuestra solidaridad en este momento de duelo, confiando en que el legado académico, ético y humano del doctor José Moreno-Montoya continúe inspirando a las generaciones presentes y futuras de los investigadores de Colombia y la región.

*Requiescat in pace.*

Bogotá, D. C., 9 de diciembre de 2025

